# Imagery use and vividness as predictors of cognitive function in collegiate athletes

**DOI:** 10.3389/fpsyg.2026.1812047

**Published:** 2026-06-08

**Authors:** Yingke Li, Nurwina Anuar, Simon Cooper

**Affiliations:** 1 Faculty of Education, Universiti Kebangsaan Malaysia, Bangi, Selangor, Malaysia; 2 Department of Sport Science, Sport Health and Performance Enhancement (SHAPE) Research Centre, Exercise and Health Research Group, Nottingham Trent University, Nottingham, United Kingdom

**Keywords:** attention, cognitive function, executive function, imagery, imagery use, imagery vividness, memory

## Abstract

Cognitive function plays an important role in how athletes regulate attention, process information, and respond to performance demands during training and competition. Collegiate athletes may be particularly reliant on effective cognitive function because they must manage simultaneous academic and athletic demands. Although imagery is widely used in sport settings, less is known about how different imagery characteristics relate to cognitive function in collegiate athletes. The present study examined the associations of imagery use and imagery vividness with cognitive function across memory, attention, and executive domains in 231 Malaysian collegiate athletes. Participants completed self-report measures of imagery use, imagery vividness, and cognitive function. Multiple regression analyses were conducted while controlling for demographic and sport-related variables. The results showed that imagery vividness was consistently associated with better cognitive function, particularly in attention and executive function, whereas imagery use demonstrated comparatively weaker associations. These findings suggest that the sensory quality and clarity of mental imagery may be more closely related to cognitive function than the frequency of imagery use alone. Overall, the study highlights the importance of distinguishing between imagery use and imagery vividness and provides further insight into the cognitive relevance of imagery in collegiate sport settings.

## Introduction

Cognitive function plays a central role in athletic performance, particularly in sports that require rapid information processing, continuous adaptation, and effective decision-making under pressure. Executive control and attentional regulation enable athletes to interpret dynamic situational cues, anticipate opponents’ actions, and adjust decisions under time constraints (Büchel et al., 2022; Marchetti et al., 2015; Vestberg et al., 2017). Memory processes further support performance by facilitating the retrieval of movement patterns, tactical strategies, and previous perceptual experiences that guide adaptive behaviour during competition (Furley and Wood, 2016). Efficient cognitive function enables athletes to sustain attentional focus, resist distraction, and coordinate goal-directed actions in fast-paced environments, capacities increasingly recognized as critical determinants of sporting success (Lepe-Martínez et al., 2020). These demands may be especially pronounced in open-skill sports, where athletes must continuously update working memory, shift attention between changing stimuli, and inhibit ineffective responses in real time.

For collegiate athletes, the cognitive demands associated with sport participation are often compounded by simultaneous academic, athletic, and social responsibilities. Unlike professional athletes, whose primary focus is typically performance preparation, collegiate athletes must balance intensive training schedules with coursework, examinations, and broader psychosocial expectations (Harper, 2009; Rutledge II, 2023). Successfully navigating these competing demands requires sustained attention, working-memory capacity, and effective self-regulation. However, these cognitive resources may be negatively affected by accumulated fatigue, stress, and training load (Meeusen et al., 2013). Research on mental fatigue has also shown that prolonged cognitive strain can impair decision accuracy, attention shifting, and inhibitory control that directly affect performance in dynamic sporting situations. Even relatively small disruptions in executive control or attentional processes may reduce reaction efficiency, impair motor coordination, and increase performance inconsistency or injury risk (Voss et al., 2010). Maintaining stable cognitive function within the demands of collegiate sport therefore represents an important challenge and highlights the need to identify psychological factors that may support cognitive performance in this population.

Imagery is widely regarded as a core psychological skill in sport and is commonly defined as the deliberate use of vivid sensory representations to simulate actions without overt physical execution (Marks, 2019; Morris et al., 2005). Within sport contexts, athletes frequently use imagery to mentally rehearse technical skills, simulate tactical scenarios, regulate emotional states, and enhance confidence (Cumming and Williams, 2012). Through repeated mental simulation, imagery may strengthen perceptual representations, reinforce movement sequences, and facilitate anticipatory processing during performance. Because imagery can be implemented without physical exertion and adapted across different training contexts, it has become one of the most widely used psychological techniques in applied sport settings.

Several theoretical perspectives provide a framework for understanding how imagery may influence cognitive function beyond performance execution alone. The PETTLEP model proposes that imagery is most effective when it closely replicates the physical, environmental, emotional, and cognitive characteristics of actual performance (Holmes and Collins, 2001). Functional Equivalence Theory further suggests that imagery engages neural processes similar to those involved during actual movement execution, indicating substantial overlap between mental simulation and physical performance (Kosslyn, 1996). From this perspective, imagery can activate neural networks associated with executive control, perceptual processing, and visual–spatial regulation (Glover et al., 2020). Within these frameworks, imagery vividness may be particularly important for cognitive function because vivid and detailed imagery more closely approximates real perceptual experience and places greater demands on attentional control, working memory, and executive regulation (Pearson et al., 2015). These perspectives collectively suggest that imagery is not only a performance-enhancement strategy but also a cognitively demanding process that may influence cognitive function through repeated activation of perceptual-cognitive mechanisms. Consistent with these theoretical perspectives, neurophysiological research indicates that imagery activates neural regions involved in movement planning, spatial processing, and cognitive control, including the supplementary motor area and premotor and parietal cortices (Mamassis and Doganis, 2004; Sheard and Golby, 2006). By engaging these networks, imagery strengthens perceptual-cognitive connections and facilitates anticipatory processing (Holmes and Collins, 2001; MacIntyre et al., 2013). Functional overlap between imagery and action execution has also been observed in studies examining motor simulation and cognitive control processes. Repeated engagement of these neural systems may enhance perceptual-cognitive processing involved in sport performance. In addition, imagery has been linked to improvements in attentional focus, self-regulation, and strategic decision-making under pressure (Cumming and Ramsey, 2009). Although these findings suggest that imagery may contribute to cognitive function, empirical research directly examining the relationship between imagery and specific cognitive domains remains relatively limited, particularly among collegiate athletes. Although these findings suggest that imagery may be associated with cognitive function, direct empirical research examining how different imagery characteristics relate to specific cognitive domains remains limited, particularly among collegiate athletes. One reason for this limitation may be that imagery has often been treated as a relatively unified psychological skill, despite evidence that imagery experiences vary across individuals in both frequency and vividness. The present study focused on two imagery characteristics: imagery use and imagery vividness. Imagery use reflects how frequently athletes engage in mental simulation during training or competition, whereas imagery vividness reflects the clarity and sensory richness of the generated images. Although related, these constructs represent different aspects of imagery experience and may differ in their relationship with cognitive function. Existing research has generally placed greater emphasis on imagery vividness, while comparatively less attention has been devoted to understanding whether imagery use itself contributes to cognitive outcomes after accounting for vividness. Examining imagery use and imagery vividness simultaneously may therefore provide a clearer understanding of whether the potential cognitive relevance of imagery depends primarily on the frequency of imagery engagement or on the imagery vividness. Cognitive function is also multidimensional. Within sport contexts, memory processes support the retention and retrieval of tactical knowledge and performance-related information, attention enables athletes to prioritize task-relevant cues while suppressing distractions, and executive control governs inhibitory control, cognitive flexibility, and working-memory updating during complex decision-making situations. These domains are highly relevant in competitive sport environments that require athletes to divide attention, adapt rapidly to changing conditions, and regulate behavioral responses under pressure. Despite their importance, relatively few studies have examined how imagery use and imagery vividness relate simultaneously to memory, attention, and executive aspects of cognitive function in athletes. Despite growing interest in the relationship between imagery and cognitive function, several important issues remain insufficiently understood. First, imagery research has rarely examined imagery use and imagery vividness simultaneously, making it unclear whether cognitive function is more strongly associated with how often athletes engage in imagery or with the clarity and vividness of the imagery experience itself. Second, previous research examining imagery and cognitive function has focused primarily on attentional processes or sport-performance outcomes, with comparatively less attention devoted to examining multiple domains of cognitive function within the same study design. As a result, it remains unclear whether imagery use and imagery vividness demonstrate similar relationships across memory, attention, and executive function. Third, relatively limited research has examined these relationships among collegiate athletes, despite this population experiencing substantial academic, physical, and psychological demands that may influence cognitive function. Collectively, these limitations highlight the need to examine imagery use and imagery vividness simultaneously in relation to multiple domains of cognitive function within collegiate athletic populations.

Accordingly, the present study examined whether imagery use and imagery vividness predict cognitive function among collegiate athletes. Cognitive function was examined across three domains: memory, attention, and executive control. Based on the theoretical frameworks and evidence reviewed above, the following hypotheses were formulated:

*H1:* Imagery use is associated with cognitive function, including memory, attention, and executive function, among collegiate athletes.

*H2:* Imagery vividness is associated with cognitive function, including memory, attention, and executive function, among collegiate athletes.

*H3:* Imagery use and imagery vividness explain additional variance in cognitive function after controlling for covariates.

By distinguishing between imagery use and imagery vividness in relation to multiple domains of cognitive function, the present study aims to clarify the distinct cognitive contributions of imagery frequency and imagery quality in collegiate athletes and to provide further insight into the cognitive role of imagery in sport contexts.

## Materials and methods

### Participants

A total of 231 Malaysian collegiate athletes voluntarily participated in this study. Eligible participants were Malaysian citizens aged 18 years or older and actively involved in competitive university-level sports. Individuals with congenital heart disease, epilepsy, acute respiratory infection, kidney disease, or other medical conditions that could affect cognitive or imagery performance were excluded. Participants also confirmed the absence of physical or mental disabilities and reported no illness or discomfort on the day of testing. All participants provided informed consent, and ethical approval was obtained from the university research ethics committee.

Participants represented a range of sports, with the largest groups from Soccer (*n* = 44), Volleyball (*n* = 11), Badminton (*n* = 14), Track and Field (*n* = 23), Frisbee (*n* = 30), Netball (*n* = 15), Hockey (*n* = 15), Basketball (*n* = 17), Rugby (*n* = 8), Softball (*n* = 13), Judo (*n* = 7), Cricket (*n* = 12), and other sports (*n* = 30). Descriptive statistics for all demographic variables are presented in Table 1.

**Table 1:** Characteristics of the subjects.

Variable	Participants		
	Overall (*n* = 231)	Males (*n* = 153)	Females (*n* = 78)
Age, years (M ± SD)	22.41 ± 0.99	22.43 ± 0.90	22.36 ± 1.19
Ethnicity, *n* (%)			
Malay	186 (80.5)	123 (80.4)	63 (80.8)
Chinese	29 (12.6)	20 (13.1)	9 (11.5)
Others	16 (6.9)	10 (6.6)	9 (5.9)
Sports type, *n* (%)			
Individual	70 (30.3)	40 (26.1)	30 (38.5)
Team	161 (69.7)	113 (73.9)	48 (61.5)
Play years, *n* (%)			
1–3	52 (22.5)	32 (20.2)	20 (25.7)
4–6	63 (27.3)	42 (27.5)	21 (26.9)
7–10	39 (16.9)	26 (17.0)	13 (16.7)
≥10	77 (33.3)	53 (34.6)	24 (30.8)
Competitive level, *n* (%)			
Expert	42 (18.2)	32 (20.9)	10 (12.8)
Proficient	56 (24.2)	45 (29.4)	11 (14.1)
Competent	80 (34.6)	47 (30.7)	33 (42.3)
Advanced beginner	40 (17.3)	23 (15.0)	17 (21.8)
Recreational	13 (5.6)	6 (3.9)	7 (9.0)
Imagery use, *n* (%)			
Always	129 (55.8)	83 (54.2)	46 (59.0)
Sometimes	98 (42.4)	69 (45.1)	29 (37.2)
Never	4 (1.7)	1 (0.7)	3 (3.8)

### Measures

#### Demographic information

Participants reported their gender, age, sports involvement, competitive level, and prior experience with imagery training.

#### Memory functioning questionnaire (MFQ)

Everyday memory performance was assessed using the 64-item MFQ, which covers four dimensions: Frequency of Forgetting, Seriousness of Forgetting, Retrospective Functioning, and Use of Mnemonics. Items are rated on a 7-point scale, with higher scores indicating better perceived memory functioning. Similarly, the MFQ exhibited high internal consistency, yielding a total *α* of 0.95 and subscale coefficients ranging from 0.77 to 0.96.

#### Divided attention questionnaire (DAQ)

Divided attention ability was measured using the 15-item DAQ (Tun and Wingfield, 1995). Each activity is rated for difficulty, perceived change, and frequency of engagement. An overall score was obtained by averaging the items, with higher scores reflecting greater difficulty. The DAQ also showed strong reliability, with an overall *α* of 0.88 and subscale values between 0.81 and 0.87.

#### Working memory questionnaire (WMQ)

Working memory difficulties were assessed with the 30-item WMQ, which provides subscale scores for Short-term Storage, Attention, and Executive Function (Tun and Wingfield, 1995). Items are rated from 0 (no problem) to 4 (very severe problem), with higher scores indicating greater difficulty. In the current sample, the WMQ demonstrated excellent internal consistency, with a total scale Cronbach’s *α* of 0.97 and subscale coefficients ranging from 0.89 to 0.91.

#### Sport imagery questionnaire (SIQ)

The 30-item SIQ (Hall et al., 1998) assesses the frequency of athletes’ use of five imagery functions across training and competition contexts. Items are rated on a 7-point scale (1 = rarely, 7 = often), with higher scores indicating more frequent imagery use. In the sample of this study, the internal consistency index of the overall scale, Cronbach’s α, was 0.99, and the α values of each dimension ranged from 0.89 to 0.95.

#### Vividness of movement imagery questionnaire-2 (VMIQ-2)

Imagery vividness was assessed using the 12-item VMIQ-2, which examines external visual, internal visual, and kinaesthetic imagery (Roberts et al., 2008). Items are rated on a 5-point scale, with lower scores reflecting more vivid imagery. Scores on the VMIQ-2 range from 1 to 5, with lower scores indicating greater imagery vividness. No reverse scoring was applied. Consequently, negative associations between VMIQ-2 scores and cognitive functioning indicate that higher vividness (lower scores) is related to better cognitive performance. The results of the current study showed that the Cronbach’s *α* coefficient of the overall scale was 0.98, and the α coefficients of the subscales ranged from 0.94 to 0.94, indicating that the scale had good internal consistency in this study.

Given participants’ English proficiency and the strong psychometric properties of the original version, the English questionnaire was used.

### Procedures

Participants were recruited through coaches and team managers, and data collection was conducted in a classroom before scheduled training sessions. After receiving standardized instructions, athletes completed the questionnaires measuring imagery characteristics and cognitive functioning. A trained researcher supervised all sessions to ensure consistency. The survey required approximately 10–15 min to complete. All questionnaires were administered in a group format in a quiet classroom setting, and responses were collected anonymously.

In this study, collegiate athletes are defined as university students registered with a sports program, with at least one year of training experience, regular participation in training activities, and involvement in inter-university, national, or international competitions.

### Data analyses

Statistical analysis of the data was conducted using IBM SPSS Statistics 29.0 (IBM SPSS Inc., Chicago, IL, USA). Confirmatory factor analyses (CFA) were performed using IBM SPSS Amos 29.0. Initial screening included checks for missing values, outliers, and normality. Reliability analyses were conducted using Cronbach’s alpha coefficients. A series of hierarchical multiple regression analyses were conducted to examine the unique contributions of imagery use (SIQ) and imagery vividness (VMIQ-2) to cognitive functioning after controlling for demographic and sport-related covariates. Covariates (gender, age, ethnicity, sport type, years of training, competitive level, and prior imagery experience) were entered at Step 1, followed by SIQ and VMIQ-2 total scores at Step 2. Separate analyses were performed for each cognitive functioning subscale. Statistical significance was set at *p* < 0.05.

Given the exclusive reliance on self-report measures, a Harman single-factor test was conducted to evaluate potential common method variance (CMV). All items from the SIQ, VMIQ-2, MFQ, DAQ, and WMQ were entered into an unrotated principal component analysis. The first component accounted for 23.45% of the total variance, indicating that no single factor explained the majority of the covariance among items. Thus, common method bias was not considered a serious threat to the validity of the findings. Although Harman’s single-factor test suggested that CMV was not a major concern, this approach has limitations, and the results should be interpreted with caution.

## Results

### Preliminary analyses

Bivariate correlations among all subscales are presented in Table 2. SIQ subscales were highly intercorrelated (range = 0.896 to 0.960), as were VMIQ-2 subscales (range = 0.972 to 0.978), supporting the use of total scores for the main regression analyses. Correlations between SIQ and VMIQ-2 subscales ranged from 0.167 to 0.324, indicating that imagery use frequency and vividness represent distinct constructs. This pattern is consistent with the multidimensional but coherent structure of imagery constructs, while the relatively low correlations between SIQ and VMIQ-2 further support their conceptual distinction.

**Table 2:** Bivariate correlations among imagery use, imagery vividness, and cognitive functioning subscales (*N* = 231).

Variable	1	2	3	4	5	6	7	8	9	10	11	12	13	14	15	16	17	18
1. SIQ_CS	—																	
2. SIQ_CG	0.960**	—																
3. SIQ_MS	0.942**	0.935**	—															
4. SIQ_MGA	0.922**	0.936**	0.896**	—														
5. SIQ_MGM	0.957**	0.948**	0.928**	0.909**	—													
6. VMIQ-2_EVI	−0.324**	−0.282**	−0.324**	−0.191	−0.275**	—												
7. VMIQ-2_IVI	−0.300**	−0.268**	−0.300**	−0.167	−0.258*	0.978**	—											
8. VMIQ-2_KI	−0.317**	−0.277**	−0.314**	−0.174	−0.268**	0.973**	0.972**	—										
9. MFQ_Freq	0.421**	0.413**	0.391**	0.373**	0.413**	−0.357**	−0.378**	−0.378**	—									
10. MFQ_Ser	0.385**	0.379**	0.364**	0.418**	0.382**	−0.158	−0.184	−0.181	0.643**	—								
11. MFQ_Ret	0.367**	0.375**	0.358**	0.336**	0.364**	−0.304**	−0.282**	−0.289**	0.454**	0.488**	—							
12. MFQ_Mne	−0.091	−0.128	−0.114	−0.107	−0.102	0.221*	0.211*	0.195	0.087	0.118	0.024	—						
13. DAQ_Dif	−0.281**	−0.297**	−0.278**	−0.241**	−0.284**	0.486**	0.493**	0.470**	−0.325**	−0.312**	−0.292**	0.173**	—					
14. DAQ_Chg	−0.239**	−0.256**	−0.210**	−0.190**	−0.235**	0.406**	0.401**	0.384**	−0.271**	−0.294**	−0.300**	0.038	0.744**	—				
15. DAQ_Tim	0.129	0.121	0.091	0.140*	0.133*	−0.261*	−0.241*	−0.269**	0.076	−0.001	0.076	−0.096	0.077	0.200**	—			
16. WMQ_Sto	−0.253**	−0.243**	−0.267**	−0.218**	−0.228**	0.611**	0.617**	0.607**	−0.393**	−0.382**	−0.372**	0.120	0.524**	0.448**	0.060	—		
17. WMQ_Att	−0.270**	−0.247**	−0.279**	−0.197**	−0.248**	0.571**	0.576**	0.553**	−0.410**	−0.363**	−0.386**	0.123	0.508**	0.402**	0.065	0.911**	—	
18. WMQ_Exe	−0.334**	−0.324**	−0.338**	−0.278**	−0.315**	0.655**	0.653**	0.647**	−0.412**	−0.402**	−0.434**	0.151*	0.531**	0.443**	0.014	0.912**	0.924**	—

N = 231. SIQ, Sport Imagery Questionnaire (CS = Cognitive Specific, CG = Cognitive General, MS = Motivational Specific, MGA = Motivational General-Arousal, MGM = Motivational General-Mastery); VMIQ-2, Vividness of Movement Imagery Questionnaire-2 (EVI = External Visual Imagery, IVI = Internal Visual Imagery, KI = Kinaesthetic Imagery); MFQ, Memory Functioning Questionnaire (Freq = General Frequency of Forgetting, Ser = Seriousness of Forgetting, Ret = Retrospective Functioning, Mne = Mnemonics Usage); DAQ, Divided Attention Questionnaire (Dif = Difficult, Chg = Change, Tim = Times); WMQ, Working Memory Questionnaire (Sto = Storage, Att = Attention, Exe = Executive). *p < 0.05, **p < 0.01. All tests two-tailed.

Confirmatory factor analyses (CFA) were conducted on all six instruments. Model fit indices are presented in Table 3. All cognitive functioning measures demonstrated excellent fit (MFQ: χ^2^/df = 1.094, CFI = 0.997, TLI = 0.996, RMSEA = 0.009, SRMR = 0.020; DAQ: χ^2^/df = 1.095, CFI = 0.997, TLI = 0.997, RMSEA = 0.010, SRMR = 0.020; WMQ: χ^2^/df = 1.159, CFI = 0.994, TLI = 0.994, RMSEA = 0.016, SRMR = 0.027). Among the imagery measures, VMIQ-2 showed excellent fit (χ^2^/df = 1.056, CFI = 0.999, TLI = 0.999, RMSEA = 0.008, SRMR = 0.020), while SIQ showed marginal fit (χ^2^/df = 2.678, CFI = 0.823, TLI = 0.805, RMSEA = 0.135, SRMR = 0.048). All standardized factor loadings were significant (*p* < 0.001). The distinct factorial structures of SIQ and VMIQ-2, combined with the low between-scale correlations, indicate that imagery use frequency and vividness are empirically distinct constructs.

**Table 3:** Model fit indices for confirmatory factor analysis.

Dependent variable	χ^2^/df	SRMR	RMSEA	CFI	TLI
MFQ	1.094	0.0197	0.009	0.997	0.996
DAQ	1.095	0.0195	0.010	0.997	0.997
WMQ	1.159	0.0265	0.016	0.994	0.994
SIQ	2.678	0.0475	0.135	0.823	0.805
VMIQ-2	1.056	0.0204	0.008	0.999	0.999

χ^2^/df = chi-square divided by degrees of freedom; CFI, Comparative Fit Index; TLI, Tucker-Lewis Index; RMSEA, Root Mean Square Error of Approximation; SRMR, Standardized Root Mean Square Residual.

Hierarchical regression analyses were conducted to examine the unique contributions of imagery use and vividness after controlling for demographic and sport-related covariates.

Assumption checks were conducted prior to the regression analyses. No substantial violations of normality, linearity, or homoscedasticity were observed. Multicollinearity diagnostics indicated satisfactory tolerance and VIF values across all predictors, and no problematic univariate or multivariate outliers were detected. Durbin-Watson values ranged from 1.75 to 2.50, indicating independence of residuals and supporting the suitability of the regression models.

### Main analysis

Hierarchical multiple regression analyses were conducted to examine whether imagery use (SIQ) and imagery vividness (VMIQ-2) predicted cognitive functioning after controlling for demographic and sport-related variables. Covariates (gender, age, ethnicity, sport type, years of training, competitive level, and prior imagery experience) were entered at Step 1, followed by imagery variables at Step 2. Results are summarized in Table 4.

**Table 4:** Hierarchical regression results for imagery use and vividness predicting cognitive function.

Dependent variable	Block 1 R^2^	Block 2 R^2^	ΔR^2^	VMIQ-2 β	SIQ β	f^2^
WMQ						
Storage	0.181	0.452	0.271***	0.577***	0.051	0.495
Attention	0.143	0.382	0.239***	0.540***	0.038	0.387
Executive	0.168	0.491	0.322***	0.607***	−0.026	0.633
DAQ						
Difficult	0.086	0.323	0.238***	0.454***	−0.162	0.352
Times	0.090	0.231	0.141**	0.345**	−0.131	0.183
Change	0.096	0.172	0.076*	−0.255*	0.096	0.092
MFQ						
Seriousness of forgetting	0.127	0.143	0.015	−0.129	0.016	0.018
Retrospective functioning	0.055	0.142	0.087*	−0.208	0.182	0.101
Mnemonics usage	0.126	0.157	0.030	0.119	−0.110	0.036
General frequency of forgetting	0.156	0.263	0.107**	−0.254*	0.178	0.145

VMIQ-2 = Vividness of Movement Imagery Questionnaire-2 (higher scores indicate lower vividness). SIQ = Sport Imagery Questionnaire (higher scores indicate more frequent imagery use). All VIF values < 2.0. f^2^ = ΔR^2^/(1 − Block 2 R^2^), Cohen’s f^2^: small ≥ 0.02, medium ≥ 0.15, large ≥ 0.35. *p < 0.05, **p < 0.01, ***p < 0.001.

Imagery substantially improved the prediction of cognitive function outcomes across all WMQ subscales. For WMQ Storage, the addition of imagery variables increased explained variance from 18.1 to 45.2% (ΔR^2^ = 0.271, *p* < 0.001). Imagery vividness emerged as a strong positive predictor (*β* = 0.577, *p* < 0.001), whereas imagery use was not significant. A similar pattern was observed for WMQ Attention, where imagery variables accounted for a significant increase in explained variance (ΔR^2^ = 0.239, *p* < 0.001), with vividness again showing a strong positive association (*β* = 0.540, *p* < 0.001). For WMQ Executive, imagery variables contributed the largest increase in variance explained (ΔR^2^ = 0.322, *p* < 0.001), with the final model accounting for 49.1% of the variance. Vividness remained a robust predictor (*β* = 0.607, *p* < 0.001), while imagery use was not significant.

Imagery variables also contributed significantly to attention outcomes. For DAQ Difficult, the inclusion of imagery variables resulted in a substantial increase in explained variance (ΔR^2^ = 0.238, *p* < 0.001), with vividness emerging as a significant positive predictor (β = 0.454, *p* < 0.001). For DAQ Times, imagery variables explained additional variance (ΔR^2^ = 0.141, *p* = 0.001), and vividness remained a significant predictor (*β* = 0.345, *p* = 0.002). For DAQ Change, the increase in explained variance was smaller but still significant (ΔR^2^ = 0.076, *p* = 0.027). In this model, vividness was negatively associated with performance (*β* = −0.255, *p* = 0.028). Imagery use was not significant in any DAQ model.

In contrast, imagery variables showed limited and inconsistent associations with memory outcomes. For MFQ seriousness of forgetting, the inclusion of imagery variables did not significantly improve model fit (ΔR^2^ = 0.015, *p* = 0.480). For retrospective functioning, imagery variables explained an additional 8.7% of variance (*p* = 0.019), though neither VMIQ-2 nor SIQ was individually significant. For mnemonics usage, imagery variables did not significantly improve model fit (ΔR^2^ = 0.030, *p* = 0.237). An exception was observed for general frequency of forgetting, where imagery variables significantly increased explained variance (ΔR^2^ = 0.107, *p* = 0.004). In this model, vividness was a significant negative predictor (*β* = −0.254, *p* = 0.020), indicating that higher imagery vividness was associated with lower reported forgetting frequency.

Across all models, imagery vividness emerged as a relatively consistent predictor of cognitive function, particularly for attention and executive processes. In contrast, imagery use did not remain significant after accounting for imagery vividness, suggesting that the vividness of imagery may be more important than its frequency.

The magnitude of the explained variance varied across cognitive domains. Relatively large proportions of variance were observed for working memory outcomes (R^2^ ranging from 0.382 to 0.491), indicating meaningful practical significance. Moderate effect sizes were found for divided attention measures, whereas smaller effect sizes were observed for several MFQ subscales. These findings suggest that imagery variables may have stronger predictive utility for working memory and attentional processes than for self-reported everyday memory function.

## Discussion

The present study examined the associations between imagery use, imagery vividness, and cognitive function across memory, attention, and executive domains in collegiate athletes. Overall, imagery vividness showed more consistent relationships with cognitive function, whereas imagery use demonstrated weaker and less stable associations. Specifically, imagery vividness was positively associated with attentional flexibility and executive control and negatively associated with reported memory difficulties. In contrast, imagery use showed limited and inconsistent predictive value across cognitive domains. Overall, imagery vividness demonstrated broader and more robust predictive value than imagery use.

One possible explanation for these findings is grounded in functional equivalence accounts of mental imagery, which propose that imagery recruits neural systems that partially overlap with those involved in actual perception and action (Kosslyn, 1996). From this perspective, the effectiveness of imagery depends on the extent to which mental simulation engages cognitive and perceptual processes similar to real performance (Glover et al., 2020). Within the sport context, the PETTLEP model provides a practical framework for applying this principle by emphasising that effective imagery should closely match the physical, environmental, task, temporal, emotional, and perspective characteristics of actual performance (Holmes and Collins, 2001). When these elements are incorporated, imagery is more likely to achieve higher levels of functional equivalence. In this framework, imagery vividness becomes particularly important because it reflects the degree of sensory and perceptual detail within the mental simulation. More vivid imagery is likely to increase the similarity between imagined and real experiences, thereby placing greater demands on attentional and working memory systems. In contrast, imagery use alone does not guarantee functional equivalence, as frequent engagement in imagery does not necessarily ensure sufficient sensory or contextual detail.

Consistent with this functional equivalence perspective, imagery vividness showed the strongest and most consistent associations with cognitive function, particularly in attention and executive domains. These cognitive processes rely heavily on top-down control mechanisms supported by fronto-parietal networks, which are also engaged during detailed mental simulation. Previous neurocognitive evidence has shown that vivid imagery is associated with increased activation in regions such as the dorsolateral prefrontal cortex and precuneus, which are central to working memory updating and attentional control (Collette and Van der Linden, 2002; Lotze and Halsband, 2006). The present findings extend this literature by suggesting that individual differences in imagery vividness are meaningfully reflected in athletes’ perceived cognitive efficiency.

The stronger associations between imagery vividness and executive and attentional function further suggest that vivid imagery is particularly relevant for cognitive processes requiring active control. Executive functions such as inhibition, shifting, and working memory updating rely on top-down regulatory mechanisms that are also engaged during detailed mental simulation (Martel and Glover, 2023). Similarly, attentional control depends on the ability to maintain and shift focus under competing demands, which may be facilitated by vivid internal representations. In contrast, the weaker associations observed for memory suggest that imagery vividness may be more closely aligned with dynamic control processes than with passive retention.

Imagery vividness also showed significant relationships with memory-related outcomes, particularly reduced forgetting and greater use of mnemonic strategies. This may reflect the role of sensory-rich representations in enhancing encoding specificity and retrieval efficiency. Imagery vividness provides more distinctive internal cues, which may strengthen memory traces and facilitate access to stored information (Cumming and Williams, 2012). However, the weaker magnitude of these effects compared to executive function suggests that imagery vividness may exert its strongest influence on higher-order cognitive control processes.

In contrast, imagery use showed limited and inconsistent associations with cognitive function. One possible explanation is that frequency of imagery engagement alone does not guarantee cognitive engagement unless sufficient sensory detail and structural clarity are present (Cumming and Williams, 2012). This interpretation is consistent with previous literature suggesting that imagery effectiveness depends not only on the frequency of imagery engagement but also on imagery quality, controllability, and ease of image generation (Cumming and Williams, 2012). However, the present study did not examine interaction effects between imagery use and imagery vividness, which represents an important direction for future research.

The present findings should also be interpreted within the context of collegiate athletes. Unlike elite athletes, collegiate athletes are required to manage simultaneous academic, athletic, and social demands. This dual-role environment increases cognitive load and mental fatigue, which may place additional pressure on attentional and executive resources (Simpson et al., 2021; Smith et al., 2021; Yongtawee et al., 2021). Within this context, imagery vividness may serve as a compensatory cognitive resource that supports attentional stability and self-regulation under competing demands.

From an applied perspective, the results suggest that interventions should prioritise improving imagery vividness rather than simply increasing imagery use. Structured approaches such as PETTLEP-based imagery scripts may enhance sensory, emotional, and contextual detail in mental simulation (Cooley et al., 2013). Training that develops multi-sensory imagery across visual, auditory, and kinaesthetic modalities may further strengthen cognitive engagement. In addition, brief monitoring of imagery vividness could be integrated into athlete support systems to identify individuals who may benefit from cognitive or psychological skills training, particularly during periods of elevated academic or training stress.

Several limitations should be acknowledged. First, the cross-sectional design precludes causal inference, and reverse causality cannot be ruled out, whereby individuals with higher cognitive function may generate more imagery vividness. Second, the study relied exclusively on self-report measures of cognitive function, which may increase the risk of shared method variance and limit construct validity. Although self-report measures provide insight into athletes’ perceived cognitive functioning in daily sport and academic contexts, they may not fully reflect objective cognitive performance. Future research would therefore benefit from incorporating performance-based cognitive assessments, such as the Stroop task, N-back task, and sport-specific decision-making paradigms, to provide a more comprehensive evaluation of cognitive function. Third, the absence of objective cognitive measures limits the ability to validate self-reported cognitive function. Fourth, the sample was restricted to Malaysian collegiate athletes, limiting generalisability. Finally, interaction effects between imagery use and imagery vividness were not examined and should be addressed in future research.

In conclusion, this study provides evidence that imagery vividness is more strongly associated with cognitive function than imagery use in collegiate athletes. The findings highlight that the quality of imagery, rather than its frequency, is the key factor linked to cognitive efficiency, particularly for executive and attentional control. Distinguishing between imagery use and imagery vividness therefore provides a more precise understanding of how imagery relates to cognitive function in sport, with important implications for both theory and applied practice.

## Conclusion

The present study examined the relationships between imagery use, imagery vividness, and cognitive function among collegiate athletes across memory, attention, and executive domains. The findings showed that imagery vividness was more consistently associated with cognitive function, particularly in attention and executive function, whereas imagery use demonstrated comparatively weaker associations. These results suggest that the clarity and sensory detail of mental imagery may be more closely related to cognitive function than the frequency of imagery practice alone. Overall, the study highlights the importance of distinguishing between imagery use and imagery vividness and suggests that imagery interventions focusing on sensory-rich and vivid mental simulation may be more beneficial for supporting cognitive function in collegiate athletes.

## Data Availability

The original contributions presented in the study are included in the article/supplementary material, further inquiries can be directed to the corresponding author.
